# Interaction of NANOS2 and NANOS3 with different components of the CNOT complex may contribute to the functional differences in mouse male germ cells

**DOI:** 10.1242/bio.20149308

**Published:** 2014-11-21

**Authors:** Atsushi Suzuki, Yuki Niimi, Yumiko Saga

**Affiliations:** 1 Division of Materials Science and Chemical Engineering, Faculty of Engineering, Yokohama National University, Yokohama, Kanagawa, Japan; 2 Department of Materials Science and Chemical Engineering, Graduate School of Engineering, Yokohama National University, Yokohama, Kanagawa, Japan; 3 Division of Mammalian Development, National Institute of Genetics, Mishima, Shizuoka, Japan

**Keywords:** Nanos, CCR4-NOT deadenylase, Germ cell

## Abstract

NANOS2 and NANOS3 belong to the Nanos family of proteins that contain a conserved zinc finger domain, which consists of two consecutive CCHC-type zinc finger motifs, and contribute to germ cell development in mice. Previous studies indicate that there are redundant and distinct functions of these two proteins. NANOS2 rescues NANOS3 functions in the maintenance of primordial germ cells, whereas NANOS3 fails to replace NANOS2 functions in the male germ cell pathway. However, the lack of a conditional allele of *Nanos3* has hampered delineation of each contribution of NANOS2 and NANOS3 to the male germ cell pathway. In addition, the molecular mechanism underlying the distinct functions of these proteins remains unexplored. Here, we report an unexpected observation of a transgenic mouse line expressing a NANOS2 variant harboring mutations in the zinc finger domain. Transcription of *Nanos2* and *Nanos3* was strongly compromised in the presence of this transgene, which resulted in the mimicking of the *Nanos2*/*Nanos3* double-null condition in the male gonad. In these transgenic mice, P-bodies involved in RNA metabolism had disappeared and germ cell differentiation was more severely affected than that in *Nanos2*-null mice, indicating that NANOS3 partially substitutes for NANOS2 functions. In addition, similar to NANOS2, we found that NANOS3 associated with the CCR4-NOT deadenylation complex but via a direct interaction with CNOT8, unlike CNOT1 in the case of NANOS2. This alternate interaction might account for the molecular basis of the functional redundancy and differences in NANOS2 and NANOS3 functions.

## INTRODUCTION

Nanos is an evolutionarily conserved protein implicated in the germ cell development of various organisms (Kobayashi et al., 1996; Subramaniam and Seydoux, 1999; Köprunner et al., 2001; Tsuda et al., 2003; Lai et al., 2011). Three Nanos homologues, *Nanos1–3*, exist in mice. NANOS3 expression begins immediately after the generation of primordial germ cells (PGCs) and continues through the migrating stages (Tsuda et al., 2003). On the other hand, NANOS2 is expressed only in germ cells colonizing male gonads (male gonocytes) and plays a key role in sexual differentiation (Tsuda et al., 2003; Suzuki and Saga, 2008). Recently, we reported that NANOS2 associates with the CCR4-NOT deadenylation complex (CNOT complex) via a direct interaction with CNOT1, which may contribute to the suppression of NANOS2-associated transcripts by RNA degradation (Suzuki et al., 2010; Suzuki et al., 2012). However, the molecular mechanisms of NANOS3 in germ cells still remain elusive, although similar functions to those of NANOS2 are predicted because of their structural similarity. In addition, we previously found that NANOS3 is up-regulated in *Nanos2*-null male gonocytes, suggesting that NANOS3 might partially substitute for NANOS2 in the differentiation of male gonocytes (Suzuki et al., 2007). However, it remains unclear whether NANOS3 plays a physiologically significant role in the absence of NANOS2, because a conditional knockout allele of *Nanos3* has not been available.

In the current study, we found that expression of a NANOS2 variant harboring mutations in the zinc finger motifs leads to suppression of NANOS3 expression in *Nanos2*-null male gonocytes, resulting in a double-null condition for NANOS2 and NANOS3. We also found that NANOS3 interacted with the CNOT complex via CNOT8 and functioned less effectively than NANOS2 in the male germ cell differentiation pathway.

## Results

### Mutations in the zinc finger domain disrupt the in vivo function of NANOS2

**Fig. 1.**
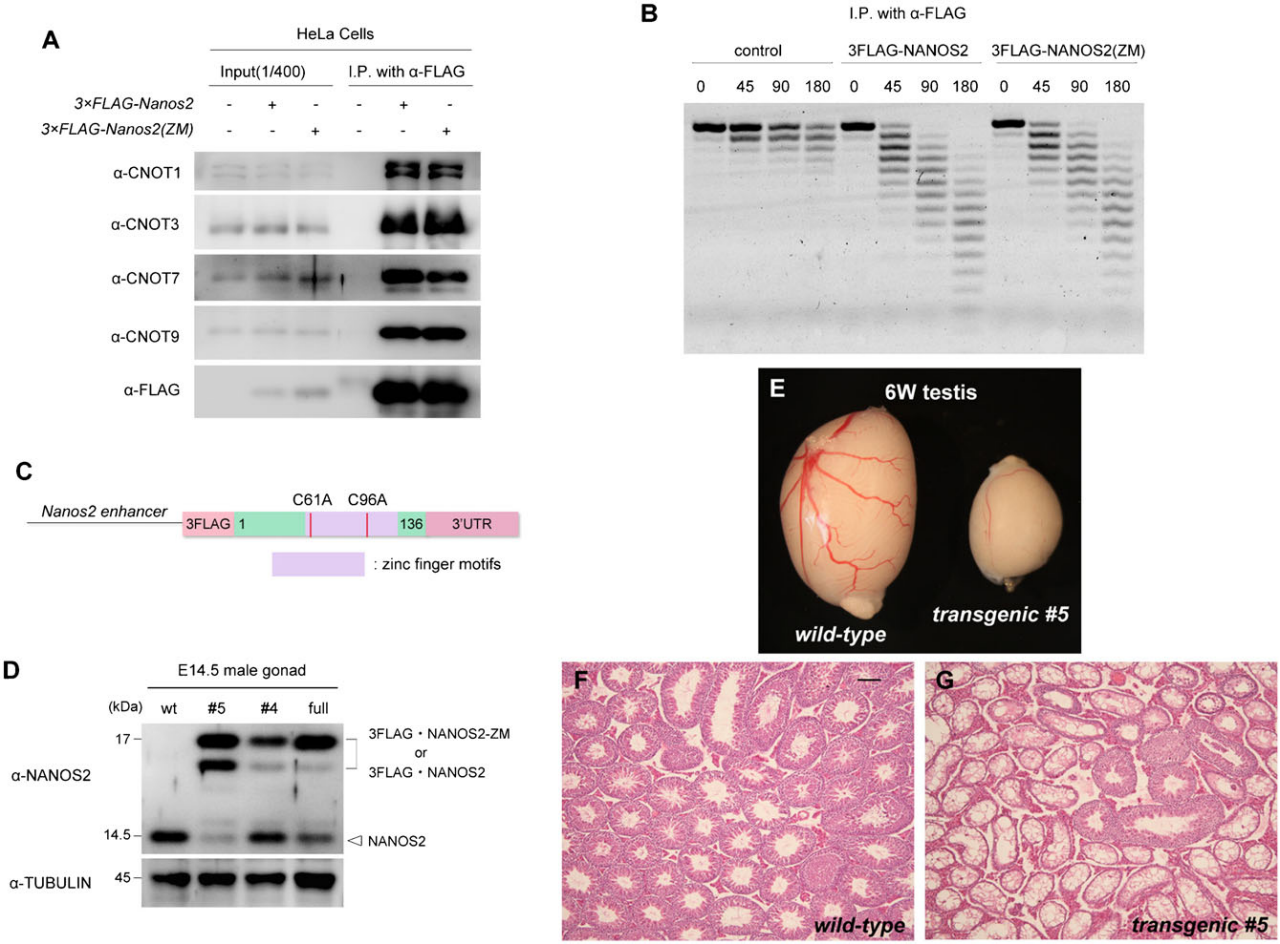
Generation of transgenic mice expressing NANOS2-ZM under the control of the *Nanos2* enhancer. (A) Flag-tagged NANOS2 or NANOS2-ZM were precipitated with anti-FLAG antibodies from HeLa cell extracts. Precipitates were analyzed by western blotting with the indicated antibodies. (B) Immunoprecipitated Flag-tagged NANOS2 or NANOS2-ZM were incubated with 5′-fluorescein isothiocyanate-labeled poly(A) RNA substrate for 0, 45, 90, and 180 minutes. Samples were then analyzed on a denaturing sequencing gel. (C) Schematic representation of the transgene encoding a NANOS2 variant harboring mutations in zinc finger motifs under the control of the *Nanos2* enhancer. (D) Western blot analysis of NANOS2 protein in E14.5 male gonads from the wild-type, Flag-tagged *Nanos2-ZM* transgenic mouse lines #4 and #5, and Flag-tagged wild-type *Nanos2* transgenic mouse line (full). Tubulin was used as a loading control. (E–G) Gross comparison (E) and hematoxylin-eosin-stained sections of testes from 6-week-old wild-type (F) and transgenic (G) mice. Scale bar, 100 µm in F for F–G.

We have previously shown that the N-terminal region of NANOS2 is required to interact with CNOT1, and this interaction is essential for NANOS2 functions in vivo (Suzuki et al., 2012). In this study, we focused on the evolutionarily conserved zinc finger domain consisting of two consecutive CCHC-type zinc finger motifs, because this domain is indispensable for in vivo functions in *Drosophila* (Lehmann and Nüsslein-Volhard, 1991). Accordingly, we generated a NANOS2 variant harboring mutations in this domain by substituting the first cysteine residues in the two CCHC motifs of NANOS2 (C61 and C96) with alanine to disrupt the structures. We refer to this NANOS2 variant as NANOS2-ZM hereafter. First, we examined whether the mutations had any effect on the interaction with the CNOT complex using HeLa cells transfected with Flag-tagged *Nanos2-ZM*. Immunoprecipitation analyses revealed that NANOS2-ZM precipitated endogenous CNOT proteins including CNOT1, which was similar to NANOS2 (Fig. 1A). In addition, and consistent with this finding, the level of deadenylase activity in NANOS2-ZM precipitates was the same as that in wild-type NANOS2 precipitates (Fig. 1B). These data indicate that the NANOS2 zinc finger domain is neither involved in the interaction with CNOT proteins nor the deadenylase activity of the CNOT complex.

Next, we investigated the physiological role of the zinc finger domain. To this end, we generated transgenic mouse lines expressing Flag-tagged NANOS2-ZM under the control of the *Nanos2* enhancer (Fig. 1C). We obtained four male (line #1–4) and one female (line #5) of transgenic founder mice. We first tried to examine expression of the transgene using embryonic male gonads derived from wild-type female mice crossed with four transgenic males (lines #1 to #4). However, we found that line #1 was sterile with small testes containing almost no germ cells (data not shown). In addition, western blotting analyses revealed no Flag-tagged NANOS2-ZM expression in the embryonic gonads derived from line #2 and #3 (data not shown). Only line #4 showed slight expression of Flag-tagged NANOS2-ZM (Fig. 1D, lane 3). On the other hand, when we crossed the female transgenic mouse (line #5) with a wild-type male mouse, male transgenic offspring had small testes with only a few germ cells (Fig. 1E–G), resulting in sterility. However, the transgene was successfully transmitted via female offspring, and eventually we could examine transgene expression in embryonic male gonads (Fig. 1D, lane 2). Therefore, we established two transgenic mouse lines, #4 and #5. Both transgenic lines produced Flag-tagged NANOS2-ZM, of which line #5 produced a higher amount (Fig. 1D, lane 2 vs. lane 3). In addition, endogenous levels of NANOS2 were almost absent in line #5, which is similar to a transgenic mouse line expressing Flag-tagged wild-type NANOS2 under the control of *Nanos2* enhancer (Fig. 1D, lane 4, full) as we reported previously (Suzuki et al., 2010). However, the level of endogenous NANOS2 was substantially higher in line #4, which might explain the difference in fertility of these two lines. Further analysis was conducted using line #5 by transmitting the transgene via female mice. We next introduced the transgene into *Nanos2*-null testes and compared the phenotype with those of *Nanos2*-null mice to examine the function of NANOS2-ZM in vivo. As shown previously, *Nanos2*-null males have significantly smaller testes than those of the wild-type, in which no germ cells exist at about 4 weeks (Tsuda et al., 2003). In this study, we also observed smaller testes in transgenic mice with a *Nanos2*-null background (supplementary material Fig. S1A), whereas a transgenic mouse line expressing Flag-tagged wild-type NANOS2 under the control of *Nanos2* enhancer fully rescue the phenotype of *Nanos2*-null mice (Suzuki et al., 2010), indicating that NANOS2-ZM has no ability to rescue wild-type NANOS2 functions. The results clearly showed that the zinc finger domain of NANOS2 is indispensable for NANOS2 functions.

### NANOS2-ZM causes disassembly of P-bodies

**Fig. 2.**
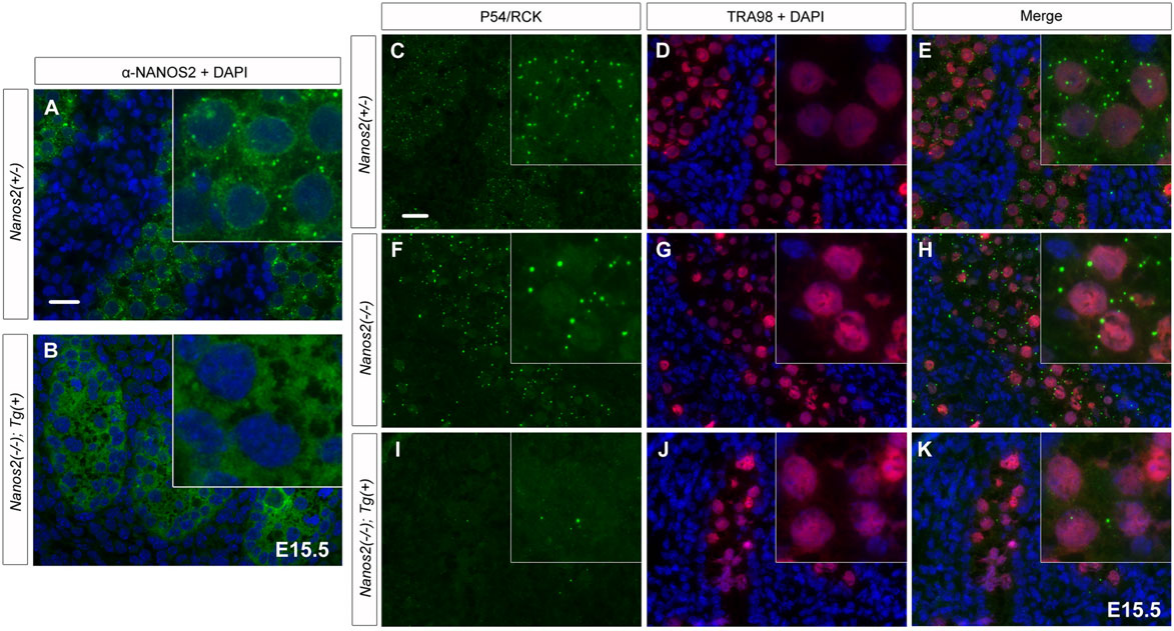
NANOS2-ZM causes disassembly of P-bodies. (A, B) Sections of E15.5 male gonads from embryos with *Nanos2*^+/−^ (A) and *Nanos2*^−/−^ with NANOS2-ZM (B) were immunostained with an anti-NANOS2 antibody (green). Scale bar, 20 µm in A for A and B. (C–K) Sections of male gonads from embryos with *Nanos2*^+/−^, *Nanos2*^−/−^, and *Nanos2*^−/−^ with the Flag-tagged NANOS2-ZM at E15.5 were immunostained with anti-p54/RCK (green) and TRA98 (red) antibodies. DNA was counterstained with DAPI (blue). Scale bar, 20 µm in C for C–K.

To further examine the properties of NANOS2-ZM, we next analyzed the cellular localization of this variant. As described previously, NANOS2 is distributed throughout the cytoplasm with some localization in discrete foci, namely P-bodies, in male gonocytes (Fig. 2A) (Suzuki et al., 2010). NANOS2-ZM was also dispersed throughout the cytoplasm, but did not localize to discrete foci (Fig. 2B), suggesting that the zinc finger domain is required for localization of NANOS2 to P-bodies. However, when we examined the status of P-bodies with an antibody against P54/RCK, a marker of P-bodies, almost no detectable discrete foci were observed in *Nanos2*-null male gonocytes expressing Flag-tagged NANOS2-ZM, whereas many cytoplasmic foci were observed specifically in male gonocytes of *Nanos2*^+/−^ and *Nanos2*^−/−^ embryos (Fig. 2I–K). These results indicate that the zinc finger domain is not required for the localization of NANOS2, but rather for the formation of P-bodies itself. Because P-bodies were obviously assembled even in the absence of NANOS2 (Fig. 2C–H) (Suzuki et al., 2010), NANOS2-ZM appears to retain negative effects on the assembly of P-bodies. In addition, this phenotype was observed in another transgenic mouse line expressing NANOS2-ZM (line #4), although expression of the transgene was restricted to a fraction of the male gonocytes in this line (supplementary material Fig. S1B–D). This observation indicated that the failure of P-body formation was caused by NANOS2-ZM, and not by the location of the transgene in the genome.

### NANOS2-ZM causes more severe phenotypes than those of *Nanos2*-knockout mice

**Fig. 3.**
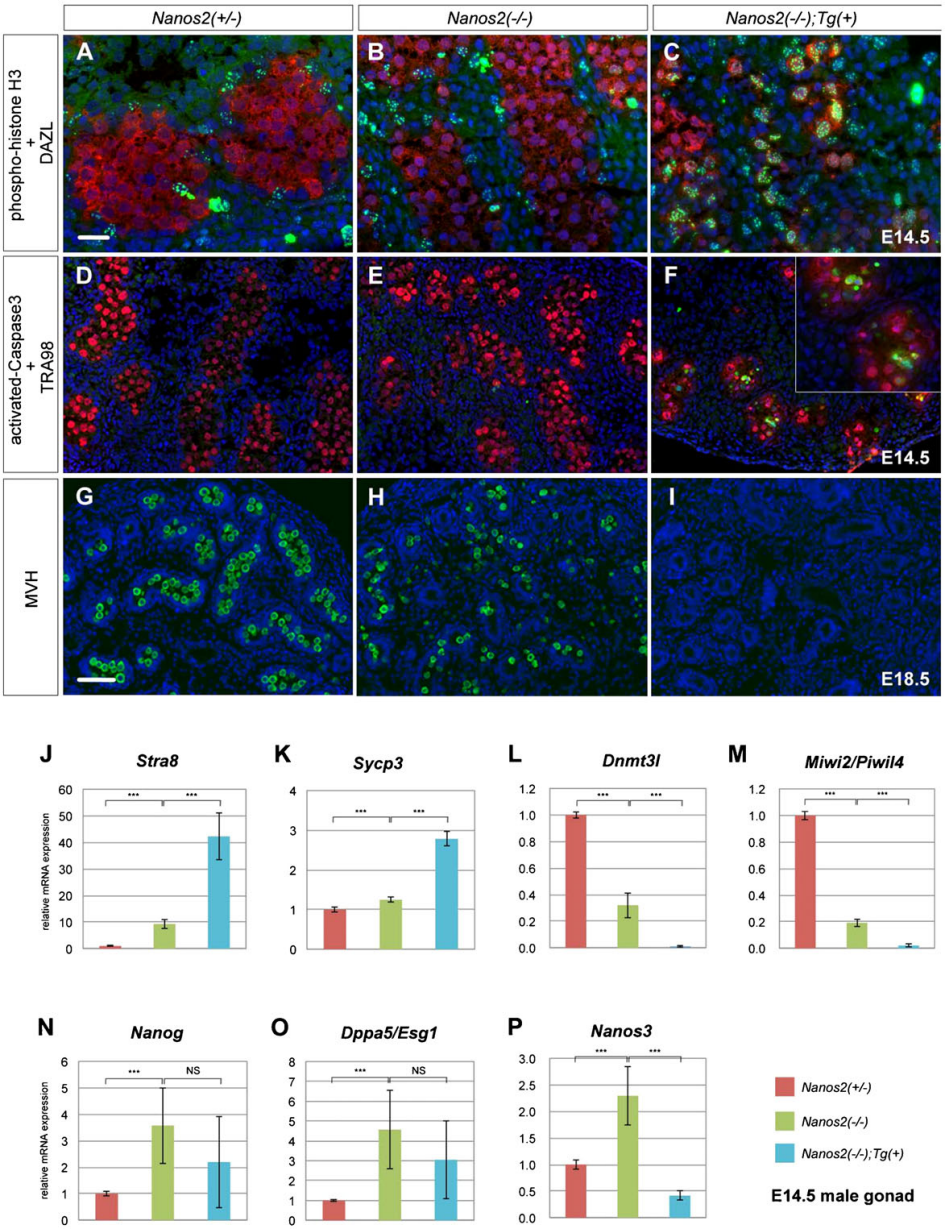
Loss-of-function of both NANOS2 and NANOS3 causes more severe phenotypes than those of *Nanos2*-null mice. (A–I) Sections of male gonads from embryos with *Nanos2*^+/−^, *Nanos2*^−/−^, and *Nanos2*^−/−^ with Flag-tagged NANOS2-ZM were immunostained with antibodies against pH3 (green) and DAZL (red) at E14.5 (A–C), activated caspase-3 (green), and TRA98 (red) at E15.5 (D–F), and MVH (green) at E18.5 (G–I). DNA was counterstained with DAPI (blue). Scale bars, 20 µm in A for A–F and 50 µm in G for G–I. (J–P) RT-qPCR analyses of the expression of *Stra8* (J), *Sycp3* (K), *Dnmt3l* (L), *Miwi2/Piwil4* (M), *Nanog* (N), *Dppa5/Esg1* (O),and *Nanos3* (P) genes in *Nanos2*^+/−^, *Nanos2*^−/−^, and *Nanos2*^−/−^ with NANOS2-ZM male gonads at E14.5. The data are shown as average relative mRNA levels±SDs (*n* = 3); ***P<0.0001 as determined by Student's *t*-test.

Because the P-body has been implicated in NANOS2-mediated RNA degradation of male gonocytes, we suspected that disassembly of P-bodies might affect the phenotype of *Nanos2*-knockout mice. Therefore, we first examined the mitotic status of male gonocytes at E14.5 by immunostaining of phosphorylated histone H3 (pH3) (Hayashi-Takanaka et al., 2009). *Nanos2*-null male gonocytes normally undergo mitotic arrest at E14.5, but fail to maintain the arrest and reinitiate proliferation from E15.5 (Suzuki and Saga, 2008; Saba et al., 2014). However, immunostaining analyses revealed that pH3 signals became apparent in the nuclei of male gonocytes from E14.5 in *Nanos2*-null mice harboring NANOS2-ZM (Fig. 3C; supplementary material Fig. S2). On the other hand, pH3-positive male gonocytes were rarely observed in *Nanos2^+/^*^−^ and *Nanos2*^−/−^ male gonads (Fig. 3A,B) as reported previously (Suzuki and Saga, 2008). This result suggests that *Nanos2*-null male gonocytes exit more quickly or never enter cell cycle arrest in the presence of NANOS2-ZM. We next examined apoptosis at E15.5 by immunostaining of activated caspase-3, because *Nanos2*-null male gonocytes frequently undergo apoptotic cell death from E16.5 (Tsuda et al., 2003; Suzuki and Saga, 2008). Consistently, activated caspase-3 signals were not observed or rare in *Nanos2^+/^*^−^ and *Nanos2*^−/−^ male gonocytes at E15.5 (Fig. 3D,E). Conversely, in the presence of NANOS2-ZM, we found drastic up-regulation of activated caspase-3 in *Nanos2*-null male gonocytes, indicating that these cells undergo more rapid apoptosis than that of *Nanos2*-null male gonocytes without NANOS2-ZM (Fig. 3F). We therefore examined the time that these cells remain viable. We have previously reported that male gonocytes survive through embryogenesis, and that some of these cells remain alive until 2 weeks after birth in *Nanos2*-knockout mice (Tsuda et al., 2003). However, immunostaining analyses with an antibody against mouse vasa homologue (MVH), a marker for germ cells, revealed no MVH-positive cells in *Nanos2*-null male gonads with NANOS2-ZM at E18.5 (Fig. 3I), although many germ cells were present in the embryonic testes of *Nanos2*^−/−^ mice (Fig. 3G,H). These data indicate that *Nanos2*-null male gonocytes disappear during embryogenesis in the presence of NANOS2-ZM, possibly because of their rapid apoptosis. Taken together, these findings suggest that the phenotypes of *Nanos2*-null male gonocytes are accelerated by the presence of Flag-tagged NANOS2-ZM.

We also examined other NANOS2 functions including suppression of the PGC character and meiosis, and promotion of male-type differentiation (Suzuki and Saga, 2008). Because each property is represented by specific gene expression (Suzuki et al., 2010), quantitative RT-PCR (RT-qPCR) was performed using total RNA from male gonads of each genotype. We first examined the mRNA expression of *Stra8* and *Sycp3*, which are implicated in meiosis (Yuan et al., 2000; Baltus et al., 2006), and found that their expression levels were much higher in the presence of NANOS2-ZM compared with those in *Nanos2*-null male gonads (Fig. 3J,K), which raises a possibility that these cells might be feminized. However, the expression level of *Stra8* in these cells was much lower than that in female gonocytes (Suzuki and Saga, 2008), and axial cores, a clear indicator of meiotic entry, were not visible (data not shown) in *Nanos2*-null male gonocytes with NANOS2-ZM possibly because of the apoptotic cell death (Suzuki and Saga, 2008), indicating that the sex of these cells was not reversed. In addition, the expression levels of *Dnmt3l* and *Miwi2/Piwil4*, markers of male-type differentiation (Sakai et al., 2004; Carmell et al., 2007; Kuramochi-Miyagawa et al., 2008), were much lower than those in *Nanos2*-null male gonads and almost absent in the presence of NANOS2-ZM (Fig. 3L,M). These data support our hypothesis that NANOS2-ZM causes the severe phenotype in *Nanos2*-null male gonocytes. On the other hand, the expression of *Nanog* and *Dppa5*/*Esg1*, markers of the PGC character (Western et al., 2005; Yamaguchi et al., 2009), were not severely changed by NANOS2-ZM in the *Nanos2*-null background, although they were up-regulated in *Nanos2*-null male gonads (Fig. 3N,O) as reported previously (Suzuki et al., 2012). We also checked the mRNA expression of *Nanos3*, presuming that it would show the same profile as that of *Nanog* and *Dppa5*/*Esg1* because of its early PGC-specific expression (Suzuki et al., 2008). However, *Nanos3* mRNA expression was severely down-regulated and had almost disappeared in the presence of NANOS2-ZM (Fig. 3P). This result raises the possibility that the disassembly of P-bodies and the severe phenotypes of *Nanos2*-null male gonocytes with NANOS2-ZM might be caused by loss of NANOS3.

### Loss of NANOS3 expression might be the primary outcome of NANOS2-ZM

**Fig. 4.**
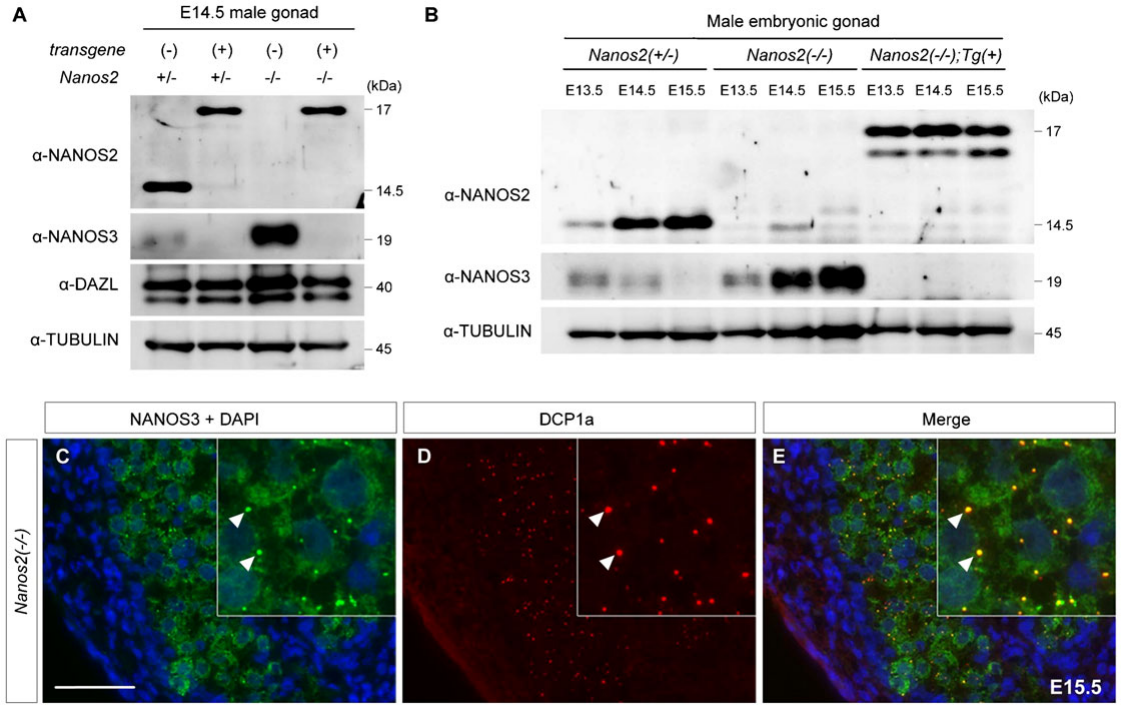
Loss of NANOS3 is the primary outcome of NANOS2-ZM. (A) Western blot analysis of E14.5 male gonads from embryos with *Nanos2*^+/−^, *Nanos2*^+/−^ with the transgene, *Nanos2*^−/−^, and *Nanos2*^−/−^ with the transgene using antibodies against NANOS2 (upper panel), NANOS3 (2^nd^ panel), DAZL (3^rd^ panel), and TUBULIN (lower panel). DAZL is a loading control for germ cells, whereas TUBULIN is a loading control for the total amount of protein. Note that DAZL is up-regulated in *Nanos2*^−/−^ male gonads as described previously (Suzuki et al., 2010). (B) Western blot analysis of male gonads from embryos with *Nanos2*^+/−^, *Nanos2*^−/−^, and *Nanos2*^−/−^ with Flag-tagged NANOS2-ZM at E13.5, E14.5, and E15.5 using antibodies against NANOS2 (upper panel), NANOS3 (middle panel), and TUBULIN (lower panel). (C–E) Sections of male gonads from *Nanos2*^−/−^ embryos at E15.5 were immunostained with antibodies against NANOS3 (green) and DCP1a (red). DNA was counterstained with DAPI (blue). Arrowheads indicate the localization of NANOS3 in P-bodies. Scale bar, 50 µm in C for C–E.

A lack of NANOS3 protein expression in the presence of NANOS2-ZM was observed in both *Nanos2*^+/−^ and *Nanos2*-null genetic backgrounds, while other germ cell proteins were less affected in the presence of NANOS2-ZM (Fig. 4A). To examine when NANOS3 expression is attenuated, we analyzed the expression profiles of NANOS3 from E13.5 to E15.5 in male gonads of each genotype (Fig. 4B). In *Nanos2*-null male gonads with NANOS2-ZM, NANOS3 expression was almost undetectable from E13.5 compared with that in *Nanos2*^+/−^ and *Nanos2*^−/−^ embryos. This finding suggested that down-regulation of NANOS3 might be the first change induced by NANOS2-ZM immediately after activation of the *Nanos2* enhancer. If the loss of NANOS3 is the primary cause for the disassembly of P-bodies in *Nanos2*-knockout mice harboring NANOS2-ZM, NANOS3 should be involved in the P-body assembly in the absence of NANOS2. Therefore, we analyzed the cellular localization of NANOS3 in *Nanos2*-null male gonocytes. Similar to NANOS2, we found that NANOS3 was dispersed throughout the cytoplasm with some localization in discrete foci (Fig. 4C). As expected, these discrete foci were almost perfectly merged with DCP1a, a marker of P-bodies, indicating localization of NANOS3 to P-bodies in *Nanos2*-null male gonocytes (Fig. 4D,E). These findings are consistent with our hypothesis that the loss of NANOS3 is the primary cause for the disassembly of P-bodies, which may be responsible for the severe phenotypes in *Nanos2*-null male gonocytes with NANOS2-ZM.

### Forced expression of NANOS3 partially rescues the assembly of P-bodies in *Nanos2*-knockout mice harboring NANOS2-ZM

We next tried to determine whether the disassembly of P-bodies and severe phenotypes in *Nanos2*-null male gonocytes with NANOS2-ZM were caused by the loss of NANOS3. For this purpose, we used our knowledge of a previously generated transgenic mouse line expressing Flag-tagged NANOS3 under the direct control of the *Nanos2* enhancer (Suzuki et al., 2007). Because this mouse line was terminated, we regenerated the transgenic mouse line and obtained three lines, of which line #1 was used for further analyses because of its high transgene expression (supplementary material Fig. S3A).

**Fig. 5.**
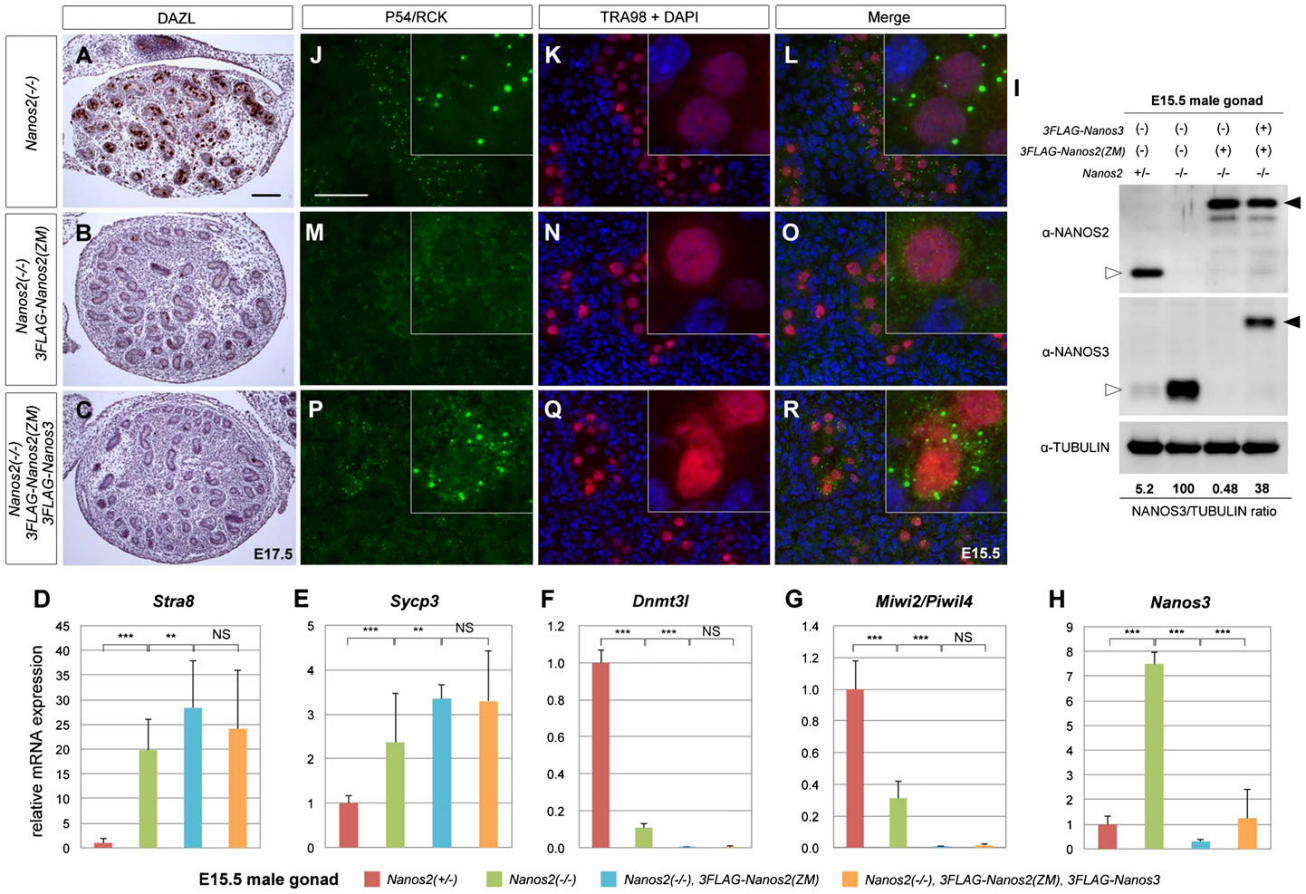
Forced expression of NANOS3 partially rescues the assembly of P-bodies in *Nanos2*-null male gonocytes with NANOS2-ZM. (A–C) Sections of male gonads from embryos at E17.5 with *Nanos2*^−/−^, *Nanos2*^−/−^ with Flag-tagged NANOS2-ZM, and *Nanos2*^−/−^ with both Flag-tagged NANOS2-ZM and NANOS3 were immunostained with an anti-DAZL antibody and then counterstained with hematoxylin. Scale bar, 100 µm in A for A–C. (D–H) RT-qPCR analyses of the expression of *Stra8* (D), *Sycp3* (E), *Dnmt3l* (F), *Miwi2/Piwil4* (G), and *Nanos3* (H) genes in *Nanos2*^+/−^, *Nanos2*^−/−^, *Nanos2*^−/−^ with Flag-tagged NANOS2-ZM, and *Nanos2*^−/−^ with both Flag-tagged NANOS2-ZM and NANOS3 male gonads at E15.5. The data are shown as average relative mRNA levels±SDs (*n* = 3); ***P<0.0001 as determined by Student's *t*-test. (I) Western blotting analysis of proteins in E15.5 male gonads from embryos with *Nanos2*^+/−^, *Nanos2*^−/−^, *Nanos2*^−/−^ with Flag-tagged NANOS2-ZM, and *Nanos2*^−/−^ with both Flag-tagged NANOS2-ZM and NANOS3 using antibodies against NANOS2 (upper panel), NANOS3 (middle panel), and TUBULIN (lower panel). The ratio of NANOS3 expression was quantified using TUBULIN for normalization. White arrowheads indicate endogenous NANOS2 or NANOS3 while black arrowheads indicate exogenous 3×Flag-tagged NANOS2 or NANOS3. (J–R) Sections of male gonads from embryos at E15.5 with *Nanos2*^−/−^, *Nanos2*^−/−^ with Flag-tagged NANOS2-ZM, and *Nanos2*^−/−^ with both Flag-tagged NANOS2-ZM and NANOS3 were immunostained with anti-p54/RCK (green) and TRA98 (red) antibodies. DNA was counterstained with DAPI (blue). Scale bar, 50 µm in J for J–R.

We introduced the transgene (line #1) into *Nanos2*^+/−^ male mice and crossed them with *Nanos2*-null female mice harboring NANOS2-ZM to obtain *Nanos2*-knockout embryos with *Flag-Nanos3*/*Flag-Nanos2-ZM* transgenes. We first examined recovery of the germ cell phenotype in *Nanos2*-null male gonocytes with NANOS2-ZM. As shown above, *Nanos2*-null male gonocytes with NANOS2-ZM disappeared at E18.5 (Fig. 3G–I). Similarly, histological analyses of *Nanos2*-null male gonads with both *Flag-Nanos3* and *Flag-Nanos2-ZM* showed that only a few germ cells had survived at E17.5 (Fig. 5A–C), indicating no recovery effects because of the *Flag-Nanos3* transgene. In contrast, we observed profound recovery of P-body assembly because of the *Flag-Nanos3* transgene at E15.5 (supplementary material Fig. S3B–J), as P-bodies were also clearly observed in *Nanos2*-null male gonocytes with Flag-tagged NANOS2-ZM when the additional transgene expressing NANOS3 was included (Fig. 5J–R). We also observed localization of exogenous NANOS3 in P-bodies (supplementary material Fig. S3H′–J′), although some of the male gonocytes exhibited no restoration of P-bodies regardless of the NANOS3 expression (supplementary material Fig. S3H″–J″). These results suggest that P-body formation is partially rescued by exogenous NANOS3.

Next, to investigate any rescue because of the recovery of P-bodies, we conducted gene expression analyses of male gonads from these embryos at E15.5. However, no rescue effects were observed with regard to the gene expression levels of meiotic (*Stra8*, *Sycp3*) (Fig. 5D,E) and male-type (*Dnmt3l*, *Miwi2/Piwil4*) (Fig. 5F,G) markers. We suspected that the apparent lack of rescue might have been due to the lower expression of NANOS3 in the presence of NANOS2-ZM. To address this possibility, we compared the expression of Flag-tagged NANOS3 among different genotypes at E15.5. Although the *Flag-Nanos3* transgene induced significant upregulation of NANOS3 (Fig. 5H, blue vs. yellow, and Fig. 5I, lane 3 vs. lane 4), its expression was still much lower than that in *Nanos2*-null male gonocytes at both the transcriptional and translational levels (Fig. 5H, green vs. yellow, and Fig. 5I, lane 2 vs. lane 4). Thus, although the small amount of exogenous NANOS3 might have been sufficient to generate substantial amounts of P-bodies, it was not sufficient for rescuing germ cell properties. These results indicate that some factor(s) affected by NANOS2-ZM other than NANOS3 might have had an effect on this severe phenotype.

### NANOS3 associates with the CNOT complex via a direct interaction with CNOT8 and shows a lower deadenylase activity than that of NANOS2

**Fig. 6.**
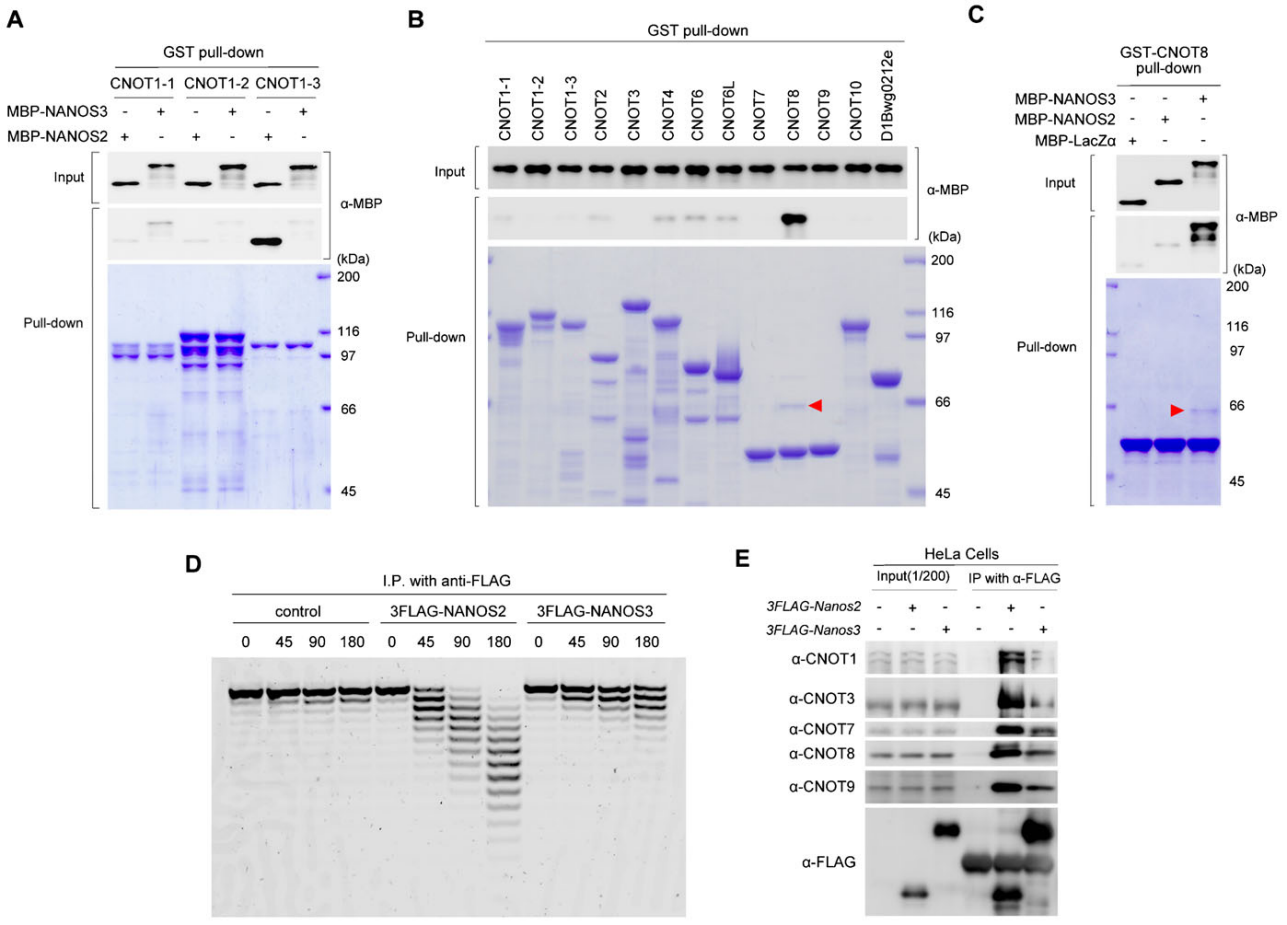
NANOS3 associates with the CCR4-NOT deadenylation complex via a direct interaction with CNOT8. (A–C) GST pull-down assay. Protein extracts from *E. coli* expressing GST-fused CNOT1-1, 1-2, 1-3, 2, 3, 4, 6, 6L, 7, 8, 9, 10, or 11/D1Bwg0212e were combined with MBP-tagged NANOS2 or NANOS3 (A). GST-fused CNOT1-1, 1-2, or 1-3 were expressed in *E coli* as described previously (Suzuki et al., 2012). Protein extracts from these *E. coli*. were combined with MBP-NANOS2 or NANOS3 (B). Protein extracts from *E. coli* expressing GST-fused CNOT8 were combined with MBP-tagged NANOS2 or NANOS3 (C). CNOT proteins precipitated with glutathione sepharose were visualized by staining with Coomassie brilliant blue, whereas co-precipitated MBP-NANOS2 or NANOS3 were detected by western blotting. Red arrowheads in (B) and (C) indicate MBP-NANOS3 co-precipitated with CNOT8. (D) Flag-tagged NANOS2 or NANOS3 were immunoprecipitated and then incubated with a 5′-fluorescein isothiocyanate-labeled poly(A) RNA substrate for 0, 45, 90, and 180 minutes. Samples were then analyzed on a denaturing sequencing gel. (E) Flag-tagged NANOS2 or NANOS3 were transfected into HeLa cells and then precipitated with anti-FLAG antibodies from protein extracts. Precipitates were analyzed by western blotting with the indicated antibodies.

In the presence of NANOS2-ZM, NANOS3 expression was strongly attenuated and accompanied by a loss of P-bodies and an enhanced *Nanos2*-null phenotype. Although exogenous NANOS3 could partly recover P-body formation, we did not observe any functional recovery. This result may be because of the low expression of exogenous *Nanos3*. However, another possible reason might be because of the different activities of NANOS2 and NANOS3 in RNA regulation. Because the functions of NANOS2 are mediated via an interaction with CNOT1 (Suzuki et al., 2012), we determined whether NANOS3 also directly associated with CNOT1. However, a GST-pull down assay did not show any positive interaction between NANOS3 and CNOT1 (Fig. 6A). We then searched for a possible binding partner of NANOS3 among CNOT proteins, which could mediate recruitment of the CNOT complex. For this purpose, GST pull-down assays were also conducted using bacterially expressed GST-fusion CNOT proteins as described previously (Suzuki et al., 2012). We found that NANOS3 only interacted with CNOT8 (Fig. 6B), while NANOS2 did not associate with CNOT8 (Fig. 6C) as shown previously (Suzuki et al., 2012), implying a different role or activity of the NANOS3-CNOT complex from that of NANOS2. We therefore compared the deadenylase activities of NANOS2 and NANOS3 by an in vitro assay system using HeLa cells. This assay revealed that cleavage of poly(A) RNA occurred intensively in NANOS2 precipitates, whereas the level of this activity was much lower in NANOS3 precipitates (Fig. 6D). In addition, western blotting of proteins co-precipitated with NANOS2 and NANOS3 in this assay showed that the amount of endogenous components of the CNOT complex (CNOT1, 3, 7, 8, and 9) was much higher in NANOS2 precipitates than that in NANOS3 precipitates (Fig. 6E). This result was consistent with the results of the deadenylase assay. These data may explain the reason why the small amount of exogenous NANOS3 in Fig. 5 had a minimal effect on the germ cell differentiation in *Nanos2*-null mice harboring NANOS2-ZM.

## DISCUSSION

Although the present study initially aimed to analyze the molecular and physiological function of the zinc finger domain of NANOS2, we unexpectedly found that mutations in the domain induced a *Nanos2*/*Nanos3* double-null condition in the *Nanos2*-knockout background. We had previously shown that NANOS2 could substitute for NANOS3 functions in the early PGC development, indicating that these structurally related proteins share similar molecular mechanisms to exert their biological functions. However, *Nanos2*-null germ cells exhibit a deleterious phenotype despite up-regulation of NANOS3. We also showed that additional NANOS3 did not recover the phenotype by overexpression of NANOS3 under the control of the *Nanos2* enhancer (Suzuki et al., 2007). We therefore presumed that NANOS3 could not replace NANOS2 functions. In the present study, we found that expression of NANOS2-ZM in *Nanos2*-null male gonocytes suppressed NANOS3 expression and resulted in more severe phenotypes than those of *Nanos2*-null male gonocytes. We suspected that this result was caused by the lack of NANOS3. However, exogenous NANOS3 could not rescue the phenotype even though P-bodies were recovered and NANOS3 was successfully recruited to the P-bodies. NANOS2-ZM was designed to lose the RNA-binding activity, while retaining sufficient activity to interact with the CNOT complex. Because some components of the CNOT complex are in the nucleus and may interact with some proteins involved in RNA transcription and epigenetic regulation (Mulder et al., 2007; Neely et al., 2010; Collart and Panasenko, 2012), this might indirectly decrease the transcription of *Nanos3*. Therefore, we could not rule out the possibility that the severe phenotypes of *Nanos2*-knockout mice harboring NANOS2-ZM were caused because of an indirect effect of NANOS2-ZM on various genes and not because of the loss of NANOS3 expression alone. Indeed, the expression of several genes was misregulated (Fig. 3J–P). Nevertheless, it can still be presumed that the expression level of NANOS3 might not have been sufficiently high or that non-functional NANOS2-ZM might compete with NANOS3 for binding to the CNOT complex. Indeed, similar to NANOS2, we showed that NANOS3 also associated with the CNOT complex, although the interaction was mediated via CNOT8, unlike CNOT1 for NANOS2, and the deadenylation activity was much lower than that of the NANOS2 complex. Therefore, it is reasonable to assume that NANOS3 is able to partially substitute for NANOS2 functions by interacting with the CNOT complex. However, NANOS3 cannot fully rescue the defects in *Nanos2*-null male gonocytes, possibly because of the weak interaction with the CNOT complex and the resultant weak deadenylase activity.

We have previously observed that NANOS2 is localized at P-bodies that are specifically formed and/or maintained in male gonocytes and have assumed that the target RNAs of NANOS2 would be degraded at these sites (Suzuki et al., 2010). However, the molecular mechanisms underlying the assembly of these structures have remained elusive. In this study, we showed that the zinc finger domain of NANOS2 was essential for P-body assembly in male gonocytes because NANOS2-ZM degraded these bodies despite that it could interact with the CNOT complex. These data suggest that the interaction of NANOS2 with its target RNA might trigger P-body assembly, whereas only its interaction with the CNOT complex is not sufficient. This proposition is consistent with the fact that the sizes and numbers of P-bodies reflect the RNA supply status (Parker and Sheth, 2007). In addition, we showed that NANOS3 might promote P-body assembly in *Nanos2*-null male gonocytes because of its interaction with the CNOT complex and its CCHC-type zinc finger domain. These data indicate that NANOS2 and NANOS3 might contribute to germ cell development via P-body assembly.

On the other hand, we found that NANOS2-ZM also decreased the expression of NANOS2 in *Nanos2*^+/−^ male gonocytes. The molecular mechanism underlying suppression of these proteins is currently unknown. We previously generated a transgenic mouse line expressing Flag-tagged wild-type NANOS2 under the control of the *Nanos2* enhancer (Suzuki et al., 2010). In this transgenic mouse, there is a significant decrease of endogenous NANOS2 mRNA, resulting in the disappearance of a substantial fraction of endogenous NANOS2 protein (Fig. 1D, lane 4) (Suzuki et al., 2010), which is similar to the case of NANOS2-ZM. Based on these data, endogenous NANOS2 mRNA might be regulated by NANOS2-ZM via the same mechanism operated by Flag-tagged wild-type NANOS2.

Based on our current results, we have devised a working model to explain the possible functional difference between NANOS2 and NANOS3 (supplementary material Fig. S4). NANOS2 interacts with all components of the CNOT complex that includes two different types of deadenylase, CNOT6 or CNOT6L (Mittal et al., 2011), and CNOT7 or CNOT8 (Aslam et al., 2009), via a direct interaction with a scaffold protein, CNOT1 (Bartlam and Yamamoto, 2010) (supplementary material Fig. S4A). On the other hand, NANOS3 would only directly form CNOT8-containing complexes (supplementary material Fig. S4B), because CNOT7 and CNOT8 appear to competitively interact with the same binding site on CNOT1 (Lau et al., 2009), which might have caused the smaller amounts of co-precipitated deadenylases with NANOS3 and the weaker deadenylase activity. The biochemical relevance of the interaction between the CNOT complex and Nanos proteins was recently investigated by Bhandari et al. (Bhandari et al., 2014). Using an in vitro binding assay, they demonstrated that all human homologues (NANOS1–3) interact with human CNOT1, which is in disagreement with the results of our GST pull-down assay. There are some sequence differences in human and mouse Nanos3, which may account for the discrepancy in results. Alternatively, different experimental conditions may have affected the results. In fact, both CNOT7 and CNOT8 were co-precipitated with NANOS3 in our experimental system using HeLa cells (Fig. 6A). We therefore cannot rule out the possibility that NANOS3 may interact with CNOT1 in vivo or with the CNOT complex via interactions with not only CNOT8, but also unknown protein(s) (supplementary material Fig. S4C).

## MATERIALS AND METHODS

### Ethics statement

Experiments were carried out with the permission of the animal experimental committee at the Yokohama National University (project number; 2014-02), which is approved April 15, 2014.

### Mice

The *Nanos2*-knockout mouse lines and PCR methods used for verification of mutant alleles have been described previously (Tsuda et al., 2003). A 3×FLAG-tagged *Nanos2-ZM* vector with a 3′-UTR under the control of the *Nanos2* enhancer (9.2 kb upstream sequence) was used for production of the transgenic mouse line. The strategy to generate transgenic mice expressing 3×FLAG-tagged NANOS3 under the control of the *Nanos2* enhancer (9.2 kb upstream sequence) has been described previously (Suzuki et al., 2007). To discriminate between Flag-tagged *Nanos2-ZM* and Flag-tagged *Nanos3*, the following primer pairs were used for genotyping: 3×Flag-tagged *Nanos2-ZM*; 3FLAG-F1, 5′-CTACAAAGACCATGACGGTG-3′, and 402-*Nanos2*-370, 5′-GACTCTGCGACCAGCTGAGTTTCGCCCACTGCG-3′; 3×Flag-tagged *Nanos3*; 3FLAG-F1, 5′-CTACAAAGACCATGACGGTG-3′, and 537-*Nanos3*-518, 5′-CTAGGCCGTAGTGGAGGGAC-3′.

### Generation of an anti-deleted in Azoospermia-like (DAZL) antibody

A 6×His-tagged DAZL fusion protein was used to immunize rabbits. Then, antibodies against DAZL were affinity-purified with GST-DAZL from anti-sera and stored in a solution of 0.1% bovine serum albumin and 0.05% NaN_3_ at 4°C. Specificity of this antibody was examined by western blotting and immunostaining (supplementary material Fig. S5).

### Immunoprecipitation and western blotting

The 3×Flag expression vectors for *Nanos2* and *Nanos3* were constructed using pcDNA3.1 (Invitrogen, USA). Details of immunoprecipitation and western blotting have been described previously (Suzuki et al., 2012). Primary antibodies against the following proteins were used in western blotting: Flag (1:8,000; F3165, Sigma), TUBULIN (1:1,000; sc-5286, Santa Cruz Biotechnology), CNOT1 (1:500; a gift from H. T. Timmers), CNOT3 (1:500; a gift from T. Tamura), CNOT7 (1:500; a gift from T. Yamamoto), CNOT8 (1:500; a gift from T. Yamamoto), and CNOT9/Rcd1 (1:500; a gift from H. Okayama).

### In vitro deadenylase assay

After immunoprecipitation, precipitates were subjected to a deadenylase assay as described previously (Suzuki et al., 2012).

### Histological methods

To immunostain DAZL and MVH, E17.5 or E18.5 male mouse gonads were fixed with 4% paraformaldehyde for 4 hours and then embedded in paraffin. After sectioning (6 µm), the samples were stained according to standard procedures. Fluorescent secondary antibodies were used for MVH, whereas horseradish peroxidase-conjugated secondary antibodies and diaminobenzidine staining were used for DAZL. To immunostain other proteins, male gonads were embedded in O.C.T. compound (Sakura) and frozen in liquid nitrogen. After sectioning (8 µm), the samples were stained according to standard procedures. Details of these methods have been described previously (Suzuki and Saga, 2008).

### GST pull-down assay

MBP-NANOS3 fusion proteins were expressed in *E. coli* BL21 (DE3) and purified with Amylose Resin (NEB). Details of these methods have been described previously (Suzuki et al., 2012).

### RT-qPCR

Total RNA isolated from male gonads was prepared using NucleoSpin® RNA XS (MACHEREY-NAGEL, Germany). Aliquots of 0.5 µg total RNA were then used for cDNA synthesis with SuperScript III reverse transcriptase (Invitrogen, USA). Quantitative PCR was performed on the Thermal Cycler Dice® Real Time System (Takara Bio, Japan) using SYBR® *Premix Ex Taq*™ II (Tli RNaseH plus) (Takara Bio, Japan) in 20 µl reactions. Each sample was analyzed in triplicate from three independent cDNA samples for each genotype. The relative amount of transcripts was calculated by the standard curve method using Multiple RQ Software (Takara Bio, Japan). Data were normalized to the endogenous reference (*G3pdh*). *Nanos2^+/−^* embryo samples were chosen to serve as the reference to normalize all other samples for each primer set. The mean and standard deviations were calculated for the triplicate measurements, and the relative target gene expression was plotted for each sample. Statistical significance was determined using the Student's *t*-test. Primer sequences are listed in the supplemental information.

### PCR primer pairs

The following PCR primer pairs were used to amplify each mRNA.

*G3pdh*-F, 5′-ACCACAGTCCATGCCATCAC-3′; *G3pdh*-R, 5′-TCCACCACCCTGTTGCTGTA-3′; *Dppa5/Esg1* for RTPCR-F, 5′-CCTTGGCAGGATGATGGTGA-3′; *Dppa5/Esg1* for RTPCR-R, 5′-CTCCAGCTTCAGCACTCCTT-3′; *Nanog*-F, 5′-AGGGTCTGCTACTGAGATGCTCTG-3′; *Nanog*-R, 5′-CAACCACTGGTTTTTCTGCCACCG-3′; *Stra8*-F, 5′-GCACATGAAGTGACACTTCC-3′; *Stra8*-R, 5′-TGGAGTGTTAACACAGCCAA-3′; *Sycp3*-F, 5′-GGTGGAAGAAAGCATTCTGG-3′; *Sycp3*-R, 5′-CAGCTCCAAATTTTTCCAGC-3′; *Dnmt3l*-F, 5′-ACTCTCCAGGTGTACACTCG-3′; *Dnmt3l*-R, 5′-TCTTCCATGCAGACACTGTC-3′; *Piwil4/Miwi2*-Fw2, 5′-TGGTCTGTGGAGATCCCATT-3′; *Piwil4/Miwi2*-Rv2, 5′-GTCCCGGTACACGACTATCC-3′; *Nanos3*-Fw, 5′-AGGGCTACACTTCTGTCTAC-3′; *Nanos3*-Rv, 5′-ATTGGATGTTGAGGCAACAC-3′.

## Supplementary Material

Supplementary Material
