# Supplementary material for: Interaction of NANOS2 and NANOS3 with different components of the CNOT complex may contribute to the functional differences in mouse male germ cells
Source: Biol Open. 2014 Nov 21;3(12):1207–16. doi: 10.1242/bio.20149308 (PMC4265758; doi:10.1242/bio.20149308)
Supplement: Supplementary Material [file supp_3_12_1207__index.html]

Interaction of NANOS2 and NANOS3 with different components of the CNOT complex may contribute to the functional differences in mouse male germ cells — Interaction of NANOS2 and NANOS3 with different components of the CNOT complex may contribute to the functional differences in mouse male germ cells — Supplementary Material 

# Interaction of NANOS2 and NANOS3 with different components of the CNOT complex may contribute to the functional differences in mouse male germ cells

## bio.20149308 Supplementary Material

**Files in this Data Supplement:**

- Supplementary Material - Atsushi Suzuki et al. doi: 10.1242/bio.20149308
