# Supplementary material for: Interaction of NANOS2 and NANOS3 with different components of the CNOT complex may contribute to the functional differences in mouse male germ cells
Source: Biol Open. 2014 Nov 21;3(12):1207–16. doi: 10.1242/bio.20149308 (PMC4265758; doi:10.1242/bio.20149308)
Supplement: Supplementary Material [file supp_bio.20149308_bio.20149308-s1.pdf]

Biology Open (2014) 000, 1–10 doi:10.1242/bio.20149308

Supplementary Material

Atsushi Suzuki et al. doi: 10.1242/bio.20149308

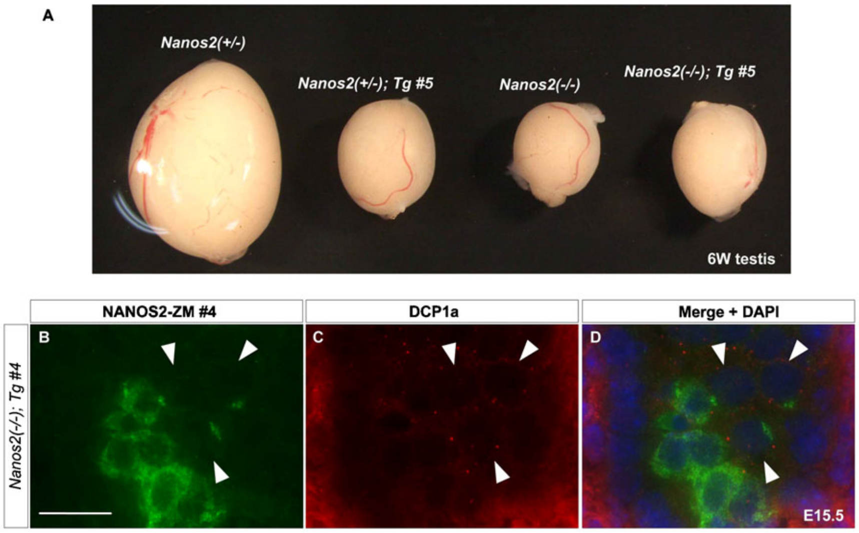

Fig. S1. NANOS2-ZM exhibits a dominant-negative effect and is unable to rescue the phenotype of *Nanos2*-knockout mice. (A) Comparison of testis size among 6-week-old mice with *Nanos2*$^{+/-}$, *Nanos2*$^{+/-}$ with the transgene, *Nanos2*$^{-/-}$ and *Nanos2*$^{-/-}$ with the transgene. (B–D) Sections of male gonads from E15.5 embryos of *Nanos2*$^{-/-}$ with Flag-tagged *Nanos2-ZM* #4 transgene were immunostained with antibodies against NANOS2 (green) and DCP1a (red). DNA was counterstained with DAPI (blue). Scale bar, 20 µm in B for B–D. Arrowheads indicate male gonocytes because P-bodies are prominently observed in these cells. Note that the assembly of P-bodies was disrupted in male gonocytes where NANOS2-ZM #5 was expressed.

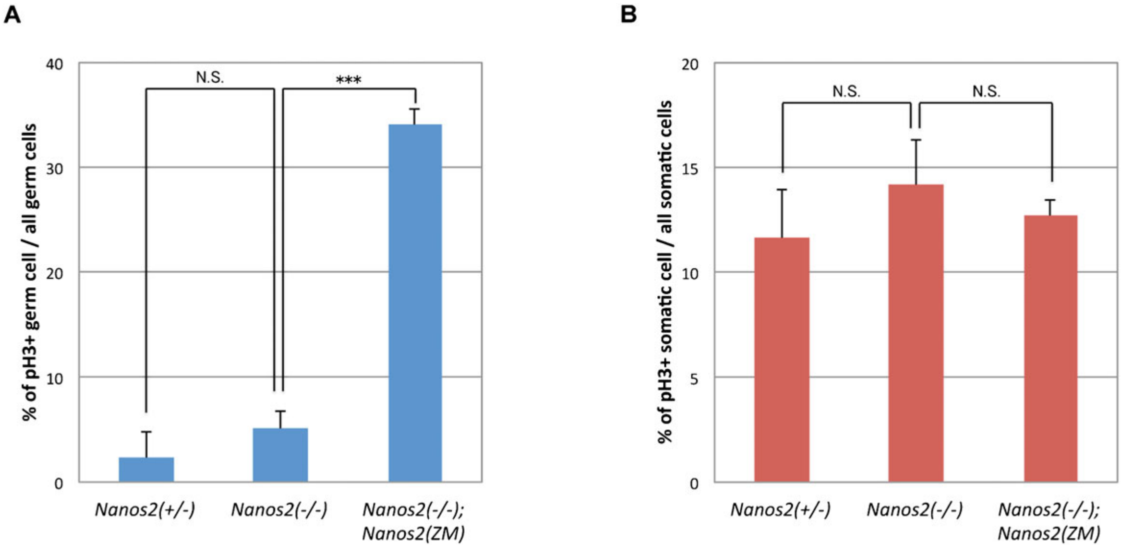

Fig. S2. The ratios of pH3-positive cells. (A,B) The ratios of pH3-positive gonocytes per all gonocytes (A) and pH3-positive somatic cells per all somatic cells (B) were calculated in male gonads from *Nanos2*$^{+/-}$, *Nanos2*$^{-/-}$ and *Nanos2*$^{-/-}$; Flag-tagged *Nanos2*-ZM transgene embryos at E14.5. The data are shown as percetages ± SDs (*n*=3); ***$P<0.0001$ as determined by Student's *t*-test..

Biology Open (2014) 000, 1–10 doi:10.1242/bio.20149308

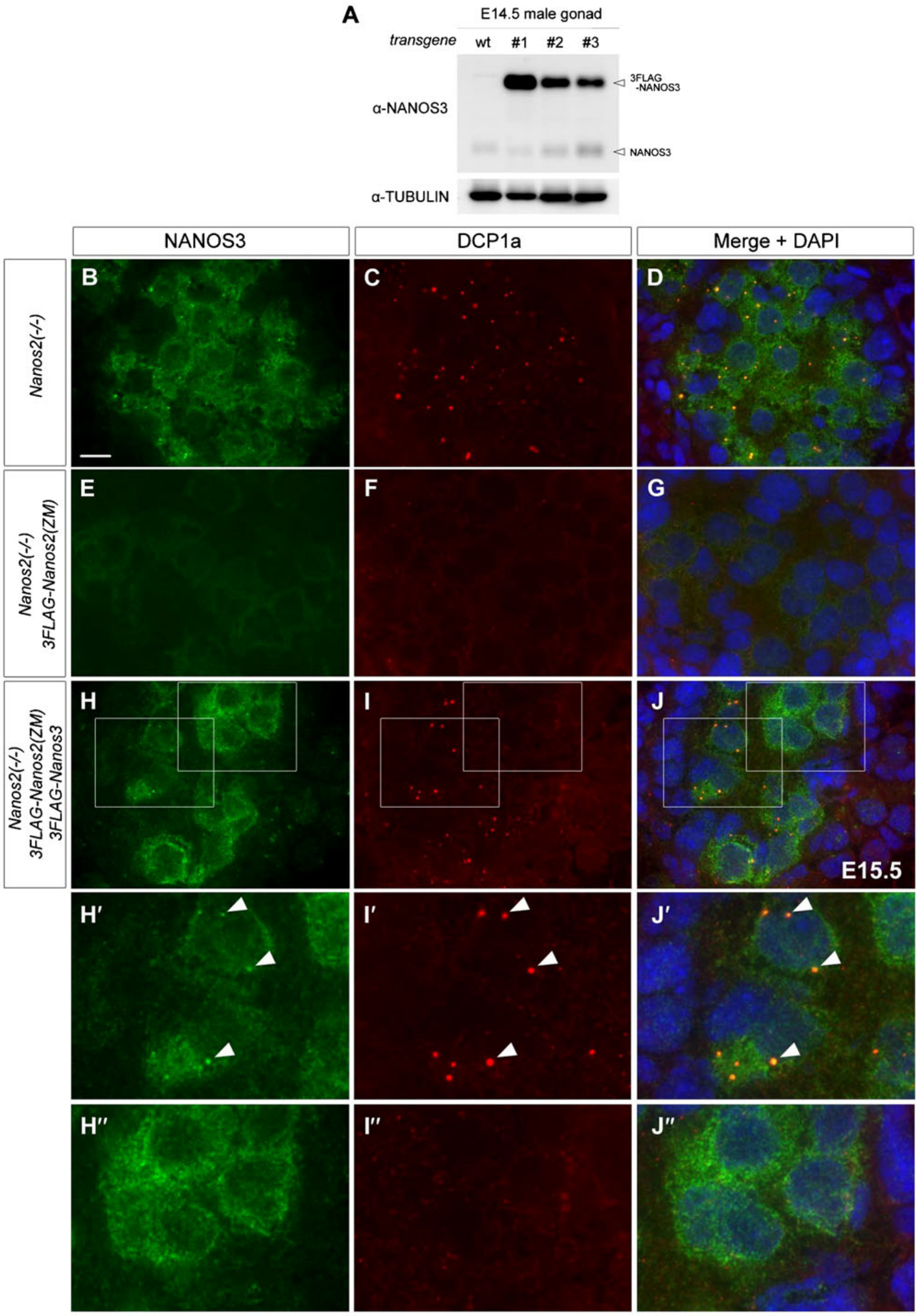

Fig. S3. Flag-tagged NANOS3 partially restores the assembly of P-body.
(A) Western blotting analysis of NANOS3 protein in male gonads from E14.5 embryos with wild-type, Flag-tagged *Nanos3* transgeneline#1, #2 and #3. Tubulin was used as a loading control. (B–J) Sections of male gonads from embryos with *Nanos2*$^{-/-}$, *Nanos2*$^{-/-}$ with the NANOS2-ZM and *Nanos2*$^{-/-}$withboth the NANOS2-ZMand the 3FLAG-NANOS3 at E15.5 were immunostained with antibodies against NANOS3 (green) and DCP1a (red). (H′–J″) High-magnification images of H–J enclosed by left squares (H′–J″) and by right-upper squares (H″–J″). Arrowheads in H′–J′ indicate colocalization of NANOS3 and DCP1a. DNA was counterstained with DAPI (blue).Scale bar: 10µm in B for B–J.

Biology Open

Biology Open (2014) 000, 1–10 doi:10.1242/bio.20149308

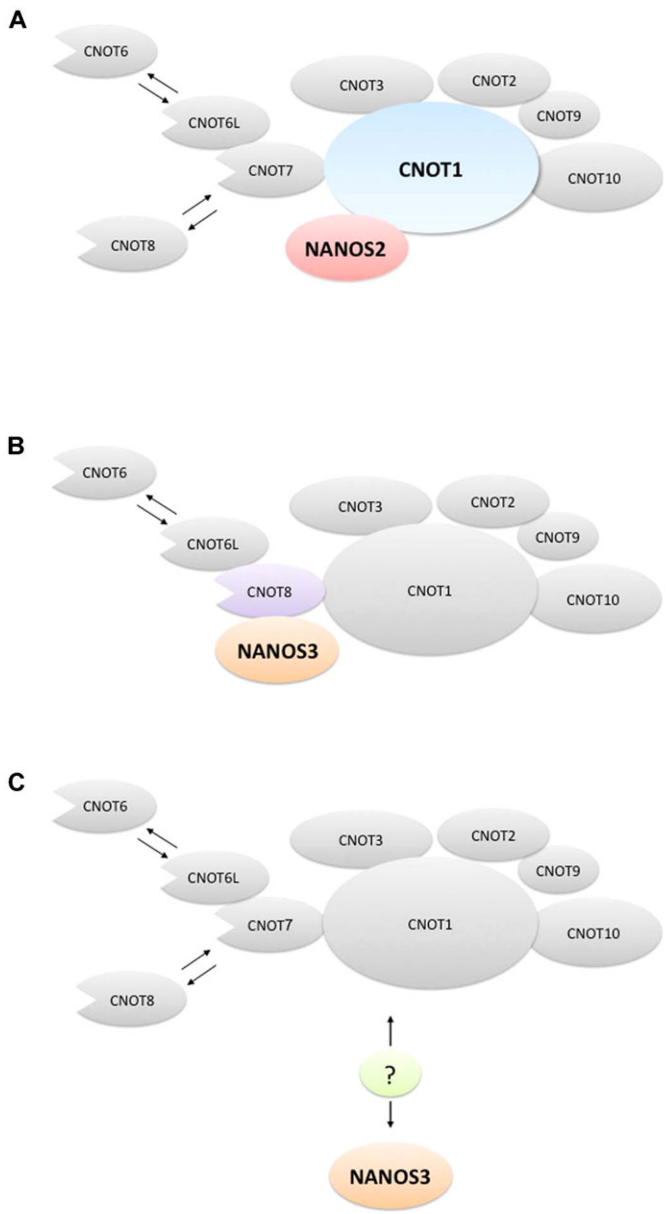

Fig. S4. Proposed models for the molecular functions of NANOS2 and NANOS3. The structure of the CNOT complex is highly conserved among eukaryotes, which consists of at least 10 CNOT proteins (CNOT1–4, 6, 6L, and 7–10) in humans and mice. Among the components of this complex, there are two different types of deadenylases. CNOT6 and CNOT6L belong to the exonuclease-endonuclease-phosphatase family, and CNOT7 and CNOT8 belong to the DEDD (Asp-Clue-Asp-Asp) family. NANOS2 can associate with all of these deadenylases via direct interaction with CNOT1 (A), whereas NANOS3 associates with the CNOT complex via interaction with CNOT8 (B), or unknown protein(s) (C), which may lead to a stronger deadenylase activity of the NANOS2 complex than that of the NANOS3 complex.

Biology Open

Biology Open (2014) 000, 1–10 doi:10.1242/bio.20149308

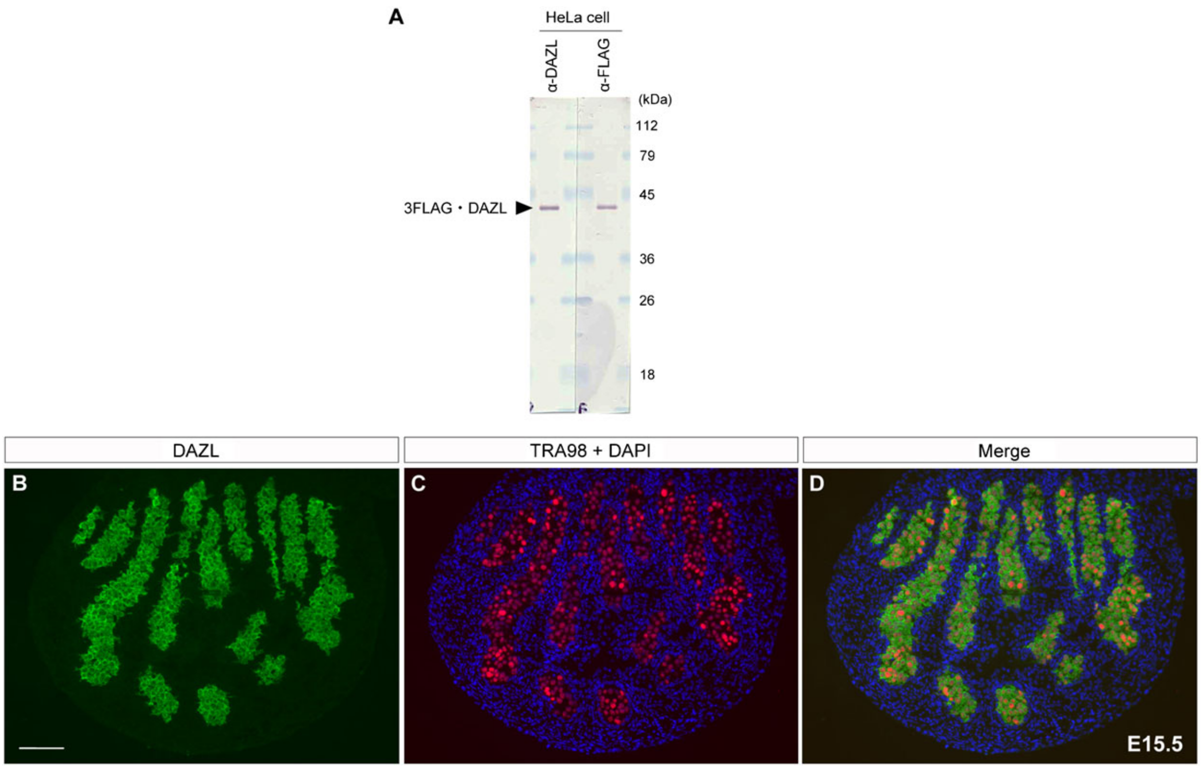

Fig. S5. Characterization of rabbit anti-DAZL antibody. (A) Western blot analysis of Flag-tagged DAZL proteins in HeLa cells after transfection with *3×FLAG-Dazl* using anti-DAZL (left lane) and anti-FLAG (right lane) antibodies. Note that both antibodies recognized a band of the same molecular weight. (B–D) Sections of male gonads from embryos at E15.5 were immunostained with antibodies against DAZL (green) and TRA98 (red). DNA was counterstained with DAPI (blue). Scale bar, 100 µm in B (for B–D). Note that the anti-DAZL antibody specifically recognized germ cells.
